# Designing Clinical Decision Support Systems (CDSS)—A User-Centered Lens of the Design Characteristics, Challenges, and Implications: Systematic Review

**DOI:** 10.2196/63733

**Published:** 2025-06-20

**Authors:** Andrew A Bayor, Jane Li, Ian A Yang, Marlien Varnfield

**Affiliations:** 1 Australian e-Health Research Centre, Commonwealth Scientific and Industrial Research Organisation, 296 Herston Rd, Herston, Brisbane, QLD, 4006, Australia, 61 7 3253 3620; 2 Thoracic Program, The Prince Charles Hospital, Brisbane, QLD, Australia; 3 Faculty of Medicine, The University of Queensland, Brisbane, QLD, Australia

**Keywords:** design, user-centered design, design challenges, emerging technologies, systematic review, integration, artificial intelligence, explainable AI, usability evaluation, design implications, implementation, clinical decision support system, CDSS, clinicians, health care delivery, diagnostics, medical conditions, patient-clinician, fast healthcare interoprabiliity resource, FHIR, electronic health records, EHRs, electronic medical records, EMRs

## Abstract

**Background:**

Clinical decision support systems (CDSS) have the potential to play a crucial role in enhancing health care quality by providing evidence-based information to clinicians at the point of care. Despite their increasing popularity, there is a lack of comprehensive research exploring their design characterization and trends. This limits our understanding and ability to optimize their functionality, usability, and adoption in health care settings.

**Objective:**

This systematic review examined the design characteristics of CDSS from a user-centered perspective, focusing on user-centered design (UCD), user experience (UX), and usability, to identify related design challenges and provide insights into the implications for future design of CDSS.

**Methods:**

This review followed the PRISMA (Preferred Reporting Items for Systematic Reviews and Meta-Analyses) recommendations and used a grounded theory analytical approach to guide the conduct, data analysis, and synthesis. A search of 4 major electronic databases (PubMed, Web of Science, Scopus, and IEEE Xplore) was conducted for papers published between 2013 and 2023, using predefined design-focused keywords (design, UX, implementation, evaluation, usability, and architecture). Papers were included if they focused on a designed CDSS for a health condition and discussed the design and UX aspects (eg, design approach, architecture, or integration). Papers were excluded if they solely covered technical implementation or architecture (eg, machine learning methods) or were editorials, reviews, books, conference abstracts, or study protocols.

**Results:**

Out of 1905 initially identified papers, 40 passed screening and eligibility checks for a full review and analysis. Analysis of the studies revealed that UCD is the most widely adopted approach for designing CDSS, with all design processes incorporating functional or usability evaluation mechanisms. The CDSS reported were mainly clinician-facing and mostly stand-alone systems, with their design lacking consideration for integration with existing clinical information systems and workflows. Through a UCD lens, four key categories of challenges relevant to CDSS design were identified: (1) usability and UX, (2) validity and reliability, (3) data quality and assurance, and (4) design and integration complexities. Notably, a subset of studies incorporating Explainable artificial intelligence highlighted its emerging role in addressing key challenges related to validity and reliability by fostering explainability, transparency, and trust in CDSS recommendations, while also supporting collaborative validation with users.

**Conclusions:**

While CDSS show promise in enhancing health care delivery, identified challenges have implications for their future design, efficacy, and utilization. Adopting pragmatic UCD design approaches that actively involve users is essential for enhancing usability and addressing identified UX challenges. Integrating with clinical systems is crucial for interoperability and presents opportunities for AI-enabled CDSS that rely on large patient data. Incorporating emerging technologies such as Explainable Artificial Intelligence can boost trust and acceptance. Enabling functionality for CDSS to support both clinicians and patients can create opportunities for effective use in virtual care.

## Introduction

Clinical decision support systems (CDSS) encompass both computerized and noncomputerized tools designed to improve and support health care delivery [1]. Computerized CDSS include sophisticated digital applications, programs, and software, while noncomputerized CDSS consist of simpler tools such as paper-based clinical support guidelines and decision aids [2]. Recently, computerized CDSS have gained significant attention due to the rapid expansion of digital technology, making them the primary focus of this review.

CDSS play a critical role in enhancing health care quality by providing reliable, accurate, and cost-efficient support for various clinical processes. Studies show that CDSS improve clinician performance [3-5], assist with medical condition management [2,6], and enhance screening procedures [7,8]. Their widespread adoption has contributed to improved patient-clinician interactions, reduced medical errors, and increased adherence to clinical guidelines [9,10].

While CDSS have demonstrated substantial impacts on health care processes, their effectiveness and overall success are intrinsically linked to their design. The way a technology is designed impacts user engagement, interaction modalities, and its ability to meet specific user needs [11,12]. These factors, in turn, shape user experience (UX), adoption rates, and perceived usefulness [13,14]. Therefore, addressing design challenges is critical to maximizing the benefits of CDSS in health care settings.

Given the complexity of health care environments and the diverse skill sets and requirements of various user groups therein, thoughtful CDSS design is essential. To achieve this, adoption of user-centered design (UCD) methodologies [15,16], which emphasize active user engagement to understand and address their unique needs throughout the design, testing, and integration phases, offers significant advantages. Through embracing such approaches, developers can create systems that function effectively and seamlessly align with user needs and expectations [17]. Thus, this review explores the design characterization of CDSS from a user-centered design perspective.

Prior review studies have explored various aspects of CDSS, including their benefits, effectiveness, adoption, and associated challenges. Research has highlighted their benefits in improving primary health care and enhancing physician performance [18-20]. Additionally, studies have examined the factors affecting CDSS adoption, identifying challenges such as privacy concerns [21], integration issues [22], and quality-in-use limitations [23].

Other studies have discussed risks and challenges, including interoperability and transportability issues [2]. In relation to the usability evaluation of CDSS, studies have revealed inconsistencies due to a lack of standardized assessment processes [24], leading researchers to propose diverse evaluation methods [25,26]. Furthermore, reviews focusing on human factors have identified challenges and barriers such as user fatigue, which can significantly impact usability and long-term adoption [27,28].

As health care systems increasingly adopt interoperability standards, which ensure that different digital systems can communicate, exchange, and use data effectively, the integration of CDSS with these advancements has become a critical area of focus. Therefore, understanding how standards such as fast healthcare interoperability resources (FHIR), digital health technologies such as electronic health records (EHRs) systems, and emerging artificial intelligence (AI) applications such as explainable artificial intelligence (XAI) integrate with CDSS is essential for effective implementation.

This review seeks to expand on existing literature by examining CDSS design through the unique lens of a UCD perspective. Recognizing the critical role of design in technology adoption, this study explores key trends in CDSS design features, the methodologies and frameworks guiding their development, and the challenges encountered in their design process. Additionally, it investigates the integration of CDSS with broader health care technologies such as electronic medical records.

Through this review, we aim to contribute to the effective design of future CDSS by examining existing challenges, proposing potential solutions, and offering recommendations. We also highlight design attributes that enhance usability, leveraging methodological trends to identify best practices. By assessing how CDSS design facilitates integration with other health systems, we provide insights into their seamless incorporation into emerging health care technologies.

## Methods

### Data Sources and Search Strategy

#### Preliminary Exploration and Refinement of Search Strategy

As an initial step in developing a comprehensive search strategy to identify all relevant papers on the design and related concepts of CDSS, we conducted a preliminary literature review and experimented with search terms. This process enabled us to establish an overarching understanding of the current knowledge landscape, refine the review’s focus, enhance the selection of search terms, and finalize the search strategy.

We selected 5 data sources, including 4 major electronic databases (PubMed, Web of Science, Scopus, and IEEE Xplore) and the *Journal of Decision Systems*, which is specific to decision support research. These sources were chosen to ensure broad coverage across health care, engineering, and design science. Related design and human-computer interaction keywords, including “user-centered design,” “design,” “user experience,” “implementation,” “evaluation,” “usability,” and “architecture,” were selected for use as search terms. Since PubMed supports MeSH (Medical Subject Headings) indexing, we also explored subject headings related to CDSS.

Based on the preliminary experimentation, we refined our selection to 4 data sources: PubMed, Web of Science, Scopus, and IEEE Xplore, due to their extensive coverage of health, engineering, and design sciences. The *Journal of Decision Systems* was excluded, as all its papers were already indexed in Web of Science and Scopus, making its inclusion redundant. Furthermore, the MeSH subheadings for CDSS did not sufficiently capture aspects related to design, usability, and UX, making MeSH-based searches in PubMed suboptimal. Consequently, we adopted 6 design-related UX and human-computer interaction keywords (“design,” “user experience,” “implementation,” “evaluation,” “usability,” and “architecture*”*) across all selected databases, including PubMed, to maintain consistency.

Regarding the scope of the search strategy, our experimentation revealed that full-text searches in PubMed and Scopus retrieved a high volume of irrelevant papers. In contrast, restricting searches to title, abstract, and keywords significantly improved specificity without omitting relevant studies, thereby reducing noise and redundancy in the search results. Based on these findings, we refined our search approach by conducting title/abstract/keyword searches in PubMed and Scopus while maintaining full-text searches in Web of Science and IEEE Xplore.

#### Data Searching and Retrieval

To retrieve relevant papers for the review, each of the 6 finalized keywords (“design,” “user experience,” “implementation,” “evaluation,” “usability,” and “architecture”) was combined with the phrase “Clinical Decision Support Systems” using Boolean operators (“AND” or “OR”), for example, “Clinical Decision Support Systems” AND “Implementation” for searching each of the 4 electronic database sources.

This comprehensive search, covering the period 2013-2023, was performed in November 2023 to identify English language papers. The identified papers were then exported using the bibliographic management software Zotero (Corporation for Digital Scholarship) [29], screened, and included in this review. The detailed search strategy is provided in Multimedia Appendix 1.

### Eligibility Criteria and Data Extraction

#### Paper Screening, Inclusion, and Exclusion

To screen and include eligible papers from the search results, the research team established predetermined inclusion and exclusion criteria (Textbox 1). Two researchers (AAB and JL) discussed and finalized these criteria, independently conducted data screening and extraction, assessed eligibility, reviewed included papers, and mapped study characteristics and findings. At each review stage, both researchers followed the same procedure in parallel, compared results, documented discrepancies, and resolved inconsistencies collaboratively. If consensus was not reached, a third researcher (MV) was consulted for a resolution.

Textbox 1. **Inclusion and exclusion criteria for the review.**

Inclusion criteria

- Paper must be written in English.
- Paper must be published between 2013 and 2023 (last 10 years).
- All paper types are eligible, except reviews, systematic reviews, and books.
- Paper must be available in full text.
- Papers based on a designed Clinical Decision Support Systems (CDSS).
- Papers that describe either the design and user experience aspects of a CDSS, such as its design approach, architecture, interfacing, or integration.

Exclusion criteria

- Papers that do not focus on a designed CDSS.
- Papers on CDSS interventions unrelated to health.
- Papers primarily focused on the technical implementation of CDSS.
- Conference abstracts and study protocols are excluded.
- Papers solely focusing on technical implementation or architecture (eg, machine learning methods).

Papers that did not center on a designed CDSS tool or intervention for a health condition or did not discuss the design aspects of the CDSS, such as its design approach, user interface, or integration, were excluded. Furthermore, papers that solely discussed the technical implementation or architecture of a CDSS, such as those focused on implementing machine learning techniques or comparing these methods, as well as editorials, scoping reviews, systematic reviews, books, conference abstracts, and study protocols, were also excluded. Textbox 1 shows the detailed inclusion and exclusion criteria.

Two researchers (AAB and JL) then independently screened abstracts to exclude ineligible papers before full-text review. A total of 130 papers were fully reviewed for eligibility based on the following: (1) whether the paper focused on a designed CDSS, (2) whether it described at least 1 aspect of CDSS design, evaluation, usability, implementation, UX, or architectural design, and (3) other criteria, including language (English), availability of sufficient information, and exclusion of conference abstracts.

After this full screening, 40 papers were included in our final analysis for the data extraction and analysis. Disparities that arose during the screening were usually discussed and resolved by the 2 researchers (AAB and JL) involved in the process, while unresolved disparities were referred to a third researcher (MV) for resolution.

#### Data Extraction

Two researchers (AAB and JL) independently reviewed all 40 included papers and conducted data extraction. The following specific categories of information were extracted from each paper and recorded in an Excel spreadsheet:

- Publication details: Title, authors, and year of publication.
- CDSS design aspects: The clinical condition addressed, design approach and methods used, UX and interaction mechanisms (ie, how users interact with the system and the type of input or output), and the intended users (clinician-facing, patient-facing, or both).
- Implementation architecture: The underlying technology of the CDSS (eg, rule-based, knowledge-based, and AI-enabled), its functional scope (eg, diagnostic support, treatment recommendation, and screening), and how it integrates with other clinical systems and workflows.
- Key findings and challenges: Insights related to the design and evaluation of the CDSS, including notable outcomes, usability concerns, and implementation barriers.

Discrepancies between the 2 researchers were discussed and resolved collaboratively. Where consensus could not be reached, a third researcher (MV) was consulted for adjudication.

### Analysis and Reporting

The data analysis and reporting of findings followed the recommendations of the Grounded Theory Literature Review (GTLR) [30] for analysis and the PRISMA (Preferred Reporting Items for Systematic Reviews and Meta-Analyses) [31] for reporting.

PRISMA is a well-documented and widely adopted guideline for reporting systematic reviews. It provides a structured checklist that ensures transparency, minimizes bias, and aligns with JMIR’s quality standards. In contrast, GTLR builds upon systematic literature reviews guidelines [32] by integrating a grounded theory approach [33], allowing key concepts to emerge inductively through thematic analysis.

According to the GTLR framework, the analytical process consists of five stages: (1) identifying the key research questions, appropriate sources, and search terms; (2) searching for potential papers; (3) defining filtering for the selection of papers and refining the sample for review; (4) a comparative and in-depth analysis of the papers through 3 coding levels; and (5) representing the emerging categories and concepts.

Given the qualitative nature of our review, combining PRISMA and GTLR was essential. GTLR enabled an in-depth, concept-driven analysis of the 40 included papers, while PRISMA ensured a systematic and unbiased selection and reporting process. Additionally, since our review was not preregistered, PRISMA helped mitigate potential reporting bias. Together, these methodologies provided a rigorous framework that balanced methodological robustness with analytical depth, ultimately enhancing the quality and reliability of our review.

For the analysis data, we followed the GTLR approach, which consists of 3 key analytical coding steps: open coding, axial coding, and selective coding [30]. Two researchers (AAB and JL) independently conducted and documented all data analyses in parallel and then compared their findings, discussed discrepancies, and resolved conflicts. Any unresolved discrepancies were escalated to a third researcher (MV) for resolution.

During the open coding step, AAB and JL independently conducted an affinity mapping of key findings from the included papers, grouping them into broad themes aligned with the primary search terms. For example, findings related to methods, usability, or evaluation were cataloged under the overarching theme of “Design Approach.” In the axial coding step, the 2 researchers independently identified and organized related subthemes within the broader themes established in the previous step.

In the selective coding step, the independently identified themes and subthemes were compared, deliberated upon, synthesized, reorganized, and validated by both researchers. These confirmed results were summarized and presented to the wider research team for further discussion. Through these collaborative and iterative processes, the review findings were refined and finalized (Multimedia Appendix 2).

## Results

### Review Characteristics

The database search yielded 1905 records from 5 sources: PubMed (279, 14.6%), Scopus (625, 32.8%), Web of Science (786, 41.3%), and IEEE Xplore (215, 11.3%). After the initial screening, 1545 records (81.1%) were removed, including 1541 duplicates (99.7%) and 4 books (0.3%), leaving 360 records for eligibility assessment.

Following the title and abstract screening, 230 records (230/360, 63.8%) were excluded. Of these, 227 (227/230, 98.8%) were removed due to irrelevant titles or abstracts, while 3 (3/230, 1.2%) were excluded as they were systematic reviews. This resulted in 130 papers for full-text eligibility review.

**Figure 1.**
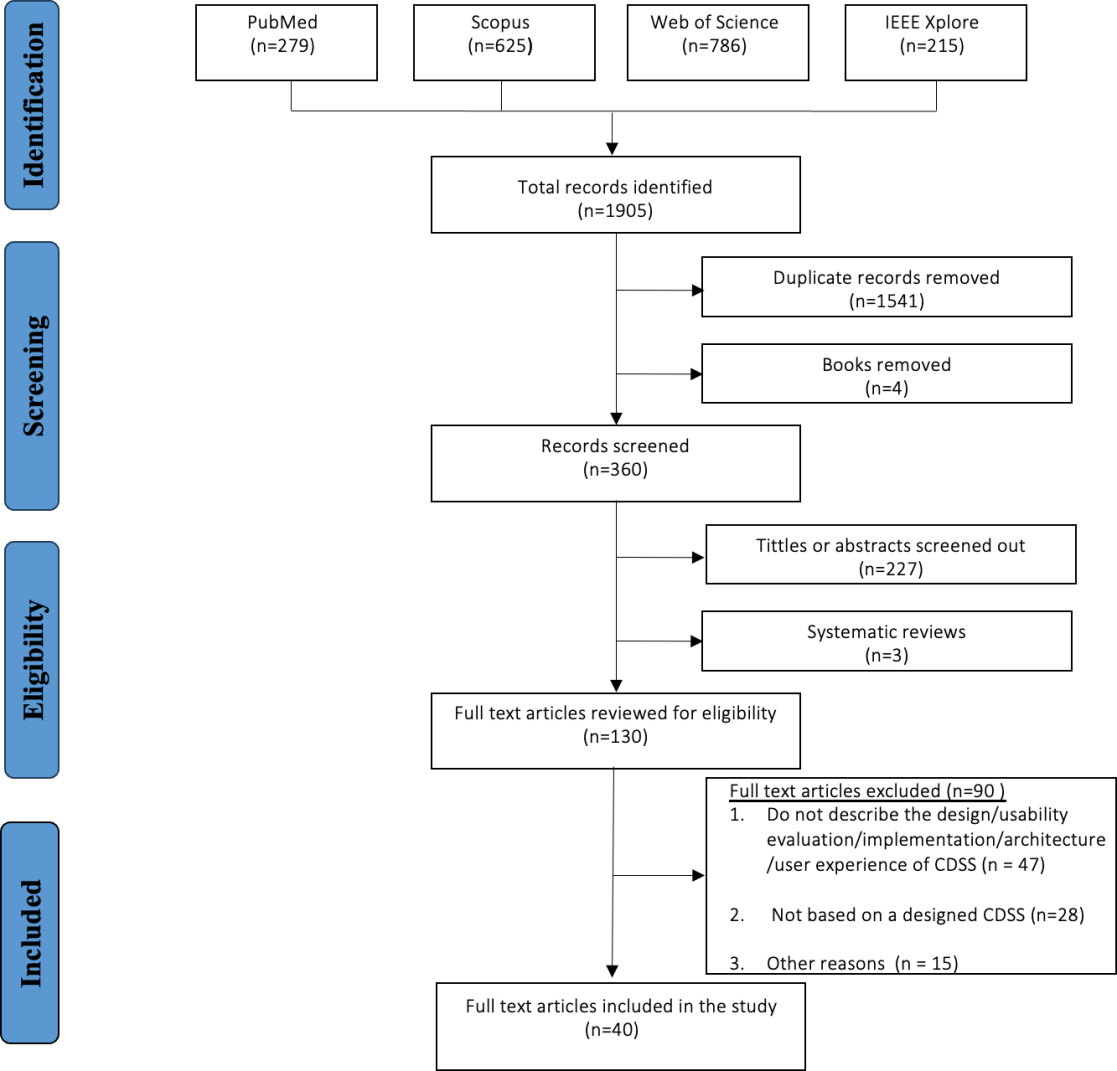
Flow diagram of the screening and selection process of included papers in the review. CDSS: clinical decision support systems.

Among these, 90 papers (69.2%) were excluded for specific reasons. Forty-seven (52.2%) lacked adequate discussion of CDSS design, evaluation, usability, implementation, architecture, or UX. Twenty-eight (31.1%) presented a CDSS that did not focus on a specific health care area or was not based on a designed CDSS. The remaining 15 papers (16.7%) were excluded due to insufficient information or because they were written in a language other than English. Ultimately, 40 papers, representing 2.1% of the original 1905 records, were included in the systematic review (Multimedia Appendix 3). Figure 1 provides a detailed overview of the paper screening and inclusion process.

### Design Characteristics

Central to this review was the identification of the design characteristics of CDSS. Accordingly, the review extracted findings from the papers, focusing on their design approach, interaction modalities, underlying technology, functional scope, and integration of the CDSS.

#### Design Approach

The review identified 4 primary approaches used in CDSS design. The UCD approach was the most common, applied in 23 out of 40 studies (57.5%). Other approaches included the knowledge-to-action framework (1/40, 2.5%), the agile business process development approach (1/40, 2.5%), and reflections based on the design of an existing system (2/40, 5%). Additionally, 13 studies (32.5%) did not explicitly reference a design approach but conducted usability evaluations or user testing.

UCD emphasizes active user involvement throughout the design process [34]. In contrast, the knowledge-to-action framework focuses on knowledge creation and translation, engaging end users at specific framework stages [35]. The agile business process development approach takes an integrated business process model to guide technology design and implementation [36].

Although UCD was the most widely used approach, its application varied across studies. Many studies using UCD involved users only in select design stages rather than throughout the entire process. Only a few studies fully adhered to the UCD framework by engaging users consistently across all phases of design [37-39]. As a result, while these studies claimed to use UCD, they often did not fully implement the approach as intended. For instance, some CDSS design stages lacked active end user participation, which is a core principle of UCD.

Across all approaches and frameworks, various data collection techniques were used, including interviews, focus groups, personas, questionnaires, and surveys. These techniques supported different aspects of the design process: informing the overall design [38,40-43], exploring user needs and workflow [44-46], contextualizing and implementing design [47-49], and ensuring usability, functionality, and effectiveness [50-52].

All CDSS examined in the reviewed papers underwent some form of usability evaluation or testing to assess functionality [42,51-54], feasibility [38,40,49], acceptability [37,38,47,49,51,52,55-58], accuracy [41,58-61]**,** quality of experience [50,62-65], and usefulness [60,65,66]. Some papers reported conducting usability testing (14/40, 37.5%), with 2 studies reporting using the System Usability Scale as an assessment tool [52,67].

Usability evaluation techniques used for data collection included think-aloud protocols, questionnaires, and interviews. The theoretical underpinnings and principles that guided the usability evaluation included the task technology fit theory, the unified theory of acceptance and use of technology, user heuristics, and the sociotechnical model. In addition, analytical tools leveraged for usability evaluation included the receiver operating characteristic curve. The design approach findings are shown in Table 1.

**Table 1.** Extract of the design approach of clinical decision support systems.

Design characteristic	Description (references)
Primary design approaches	User-centered design focus [37-40,43-49,52,55,57-59,61,63,65,68-71]. Knowledge-to-Action framework [41]. Agile Business Process Development Approach [42]. Designed reflecting on an existing CDSS [53,72]. No design approach was stated, but usability evaluation or usability testing of the system was conducted [50,51,54,56,60,62,64,66,67,73-76].
Data-gathering techniques	Interviews [41,43,44,59,65,68,70,71]. Focus groups [38,41,52,55,73]. Personas [40]. Questionnaires [41,46,55,65,68]. Surveys [37,50,57,73]
Usability testing or evaluation	Conducted usability testing [38,40,41,52,53,55,59,65-68,71-73,76]. Conducted system usability evaluation [37-70,72-76].
Usability evaluation techniques	Think Aloud protocol [41,52]. Used the System Usability Scale [52,67].
Usability evaluation theories	Unified Theory of Acceptance & Use of Technology [55]. Sociotechnical model [44]. User Heuristics [39,40]. Task Technology Fit Theory [37].
Usability evaluation analysis	Receiver operating characteristic (ROC) curve [59].

#### User-Facing Roles

**Figure 2.**
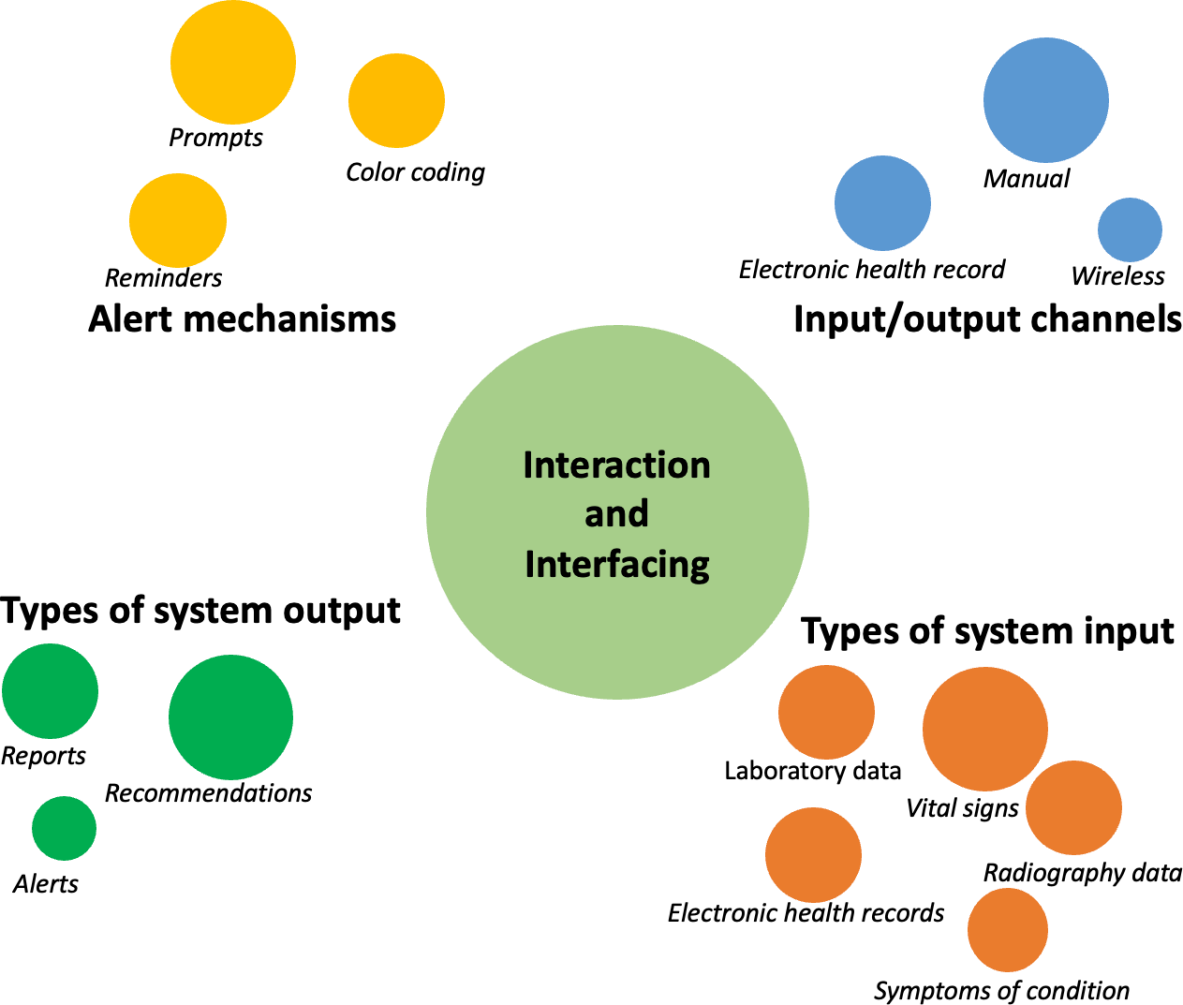
Interaction and user-facing characteristics of clinical decision support systems.

The review also examined user interaction and engagement with CDSS, delineating the design mechanisms used for input and output access, as well as for alerting and notifying users, as illustrated in Figure 2. The primary users of CDSS in the reviewed studies were predominantly clinicians (37/40, 92.5%), with only 3 systems (3/40, 7.5%) designed for both clinicians and patients.

Primarily, CDSS uses 3 channels for data or information access and input. The first is manual entry, where users input necessary data or request recommendations [38,40,41,44,46-49,55,56,58,59,64,65,67,69]. The second is integration with EHR, where input or output data are automatically retrieved by an existing EHR system integrated with the CDSS [37,42,44,45,49,52-55,60,61,64,69-71,74]. The third is wireless transmission, where input data are sourced from other devices such as sensors via Bluetooth or similar wireless data transmission protocols [53].

The types of input data and information required by CDSS for making decisions encompassed combinations of 5 categories of data sources: vital signs data such as temperature, blood pressure, and heart rate [38,46,47,54,58,62,63,65,66]; laboratory data (test results) [41,51,53,56,59,66,73,76]; EHRs (specific or combinations of information such as clinical history, comorbidities, or prescriptions) [37,42,44,45,49,52-55,60,61,64,71,74]; radiography data (results of image scans) [59]; and symptoms of condition (specific condition–related vital symptoms, eg, signs of inflammation for fractures) [40,46,50,56,62,67,69,75].

As output, systems deliver recommendations on treatment plans, workflow, or dosage [49,54,64,74]; reports on conditions including providing explanatory education on the situation and recommending options [41,53,54,59,65,68]; and alerts as feedback [41,51,53,55,67]. To facilitate the effective relay of output feedback, ensure visibility, engagement, and adherence; various alert and notification mechanisms, including prompts such as beeps or pop-ups [46,53,55,67], color coding of recommendations to signify severity [40,41,51], and sending messages as reminders [55,64], are used.

#### Underlying Design Architecture

All CDSS reported in the reviewed papers were knowledge-based, classified according to their underlying knowledge, reasoning, or support structures. No non–knowledge-based architectural designs were identified. These systems follow 1 of 4 reasoning architectures: rule-based, machine learning-based, case-based, or ontology-based. Rule-based systems use deterministic “if-then-else” rules for decision-making [77]. Machine learning–based systems develop knowledge from existing clinical data to apply it to new datasets [78,79]. Case-based reasoning systems compare new cases with stored past decisions to identify similarities and determine outcomes [80]. While ontology-based, leverage semantic web technologies to represent medical concepts and support decision-making [81]. However, there was a notable absence of ontology-based reasoning systems among the reviewed papers, likely due to the emerging nature of this category of CDSS [81].

Also, a significant trend observed in the review was the increasing use of AI and machine learning–based approaches to develop predictive decision support systems [39,43,47,48,53,57,60,61,73,75]. These AI-driven systems aim to enhance diagnostic accuracy and provide personalized recommendations based on data patterns and historical cases.

Functionally, the CDSS reviewed in the papers fall into 3 main categories: diagnostic systems, which are used for screening and identifying health conditions; treatment systems, which provide recommendations and guidance on treatment options; and dosage prescription systems, which assist in determining the appropriate dosage or combinations of drugs and treatments. The design architecture and functional scope of the reviewed CDSS are summarized in Table 2.

**Table 2.** Underlying architecture and functional scope of clinical decision support systems (CDSS) design.

Design characteristic	Description (references)
Knowledge classification	Knowledge-based: all CDSS in the reviewed papers classified as knowledge-based. Non–knowledge-based: none of the CDSS in the reviewed papers classified as non–knowledge-based.
Reasoning classification	Rule-based [39,43,47,48,53,57,60,61,67,73,75]. Case-based [58,67]. Machine learning or AI^a^-enabled [39,43,47,48,53,57,60,61,73,75].
Support classification	Diagnostic [40,45,48-51,54-59,61,64,66-69,71,73,74,76]. Treatment [40,43,45-47,49,50,53,62,63,65,67,69-71,75]. Dosage prescription [38,46,54,60,65]. Education or discussion [41,47,50,52,53,67].

a AI: artificial intelligence.

A notable development among some CDSS is the integration of interactive, social media–like modules that allow clinicians to share information, engage in discussions, and enhance their knowledge on supported health conditions. These collaborative features promote peer-to-peer learning and real-time knowledge exchange, enhancing the overall effectiveness of the CDSS [41,47,50,52,53,67].

#### Integration With Existing Health Records Systems

An important and emerging trend in the design of reviewed CDSS is their integration with other clinical systems. We assessed whether these systems were integrated with existing digital health technologies such as EHR systems, or whether they were explicitly designed with integration in mind. This assessment is critical, given the recent shift toward the adoption of emerging digital health technologies and the development of interoperability standards such as FHIR and architectural platforms such as the Substitutable Medical Applications and Reusable Technologies [82].

Our findings indicate that most CDSS were stand-alone systems, with only 20% (8/40) of the CDSS reviewed being integrated into EHRs and existing workflows, and 17.5% (7/40) of them being intentionally designed with integration as a core feature [37,42,44,45,49,52-55,60,61,64,71,74]. However, a significant portion of the CDSS reported in the reviewed papers lacked integration considerations in their design.

### Design Challenges

As an integral aspect of this review, we identified and mapped the associated design challenges of the CDSS discussed in the reviewed papers. The fundamental issues identified were categorized into the following broader themes: usability and UX challenges, validity and reliability concerns, data quality and assurance issues, and design and integration complexities, as elaborated in Table 3.

**Table 3.** Design challenges of clinical decision support systems.

Design challenge	Description (references)
Usability or user experience (UX) challenges	Advisory restrictions and inflexibilities [39,46,70]. Workflow, tailoring, and integration issues [42,49,51,55,62,64,76]. Information overload and user fatigue concerns [40,55,60,70,76]. Visual and explainable preferences [39,43,61,65,75]. Covert surveillance concerns [55]. Support issues [38,49,75].
Validity or reliability concerns	Validation challenges [54,60,64,71]. Trust and reliability issues [39,44,56,61,71,72]. Conflicting recommendation with expert opinion [45,48,54,74]. Recommendation ambiguity [46,47,65].
Data quality or assurance issues	Data quality, extraction, and access challenges [41,47,53,67]. Interoperability challenges [39,54,71].
Design or integration complexities	Transforming guidelines decision rules [41,54,55]. Contextual and integration challenges [37,39,66,68,71,74].

#### Usability or UX Challenges

A fundamental design challenge of CDSS pertains to UX and expectations. Users expressed concerns regarding how recommendations are presented and accessed [46] and inconsistencies in workflow integration [49,62]. Several studies highlighted limitations in the presentation design of CDSS recommendations, noting rigid structures that restrict users from incorporating their own perspectives [45,46,70,74]. As a result, users may feel obligated to follow system recommendations without the flexibility to adjust or choose from alternative suggestions.

Additionally, challenges arise in users’ ability to appropriately apply CDSS recommendations, prompting the need for visual aids such as video explanations to clarify their application and reduce ambiguity [46,47,65]. Also, information overload was another prevalent concern, as users may experience fatigue from the constant prompts, notifications, or input requests generated by CDSS [40,55,76].

Moreover, the design of CDSS to track usage and activity logs can evoke feelings of covert surveillance, impacting UX [55]. Furthermore, a mismatch between users’ practical workflow and the requirements of CDSS poses significant challenges [42,49,51,62,76]. For instance, CDSS may demand information that is available only several steps ahead in the operational workflow, creating a disconnect between user practices and system functionality [49,51]. Finally, other UX-related issues identified included requirements for training and technical support in the deployment and use of CDSS [38,49,75].

#### Validity or Reliability Concerns

A critical challenge concerning CDSS revolves around the attributes of their trustworthiness. Key fundamental challenges related to their validation, including determining where to initiate the validation process, establishing a “ground truth” for validation, and defining a criteria for success in validation [54,60,64,71]. For instance, how should contextual factors be factored in, and how can learning be incorporated into the validation process? Moreover, what constitutes success: tangible improvements in patients’ clinical outcomes or the functional effectiveness of the underlying technology? If the former, how can other contextual behavioral contributors be modeled to validate success?

Another significant is ambiguity in recommendations. Some terminologies used in CDSS recommendations may be unfamiliar or interpreted differently by users, leading to potential miscommunication [46,47,65]. Furthermore, trust issues arise, as more experienced clinicians may disregard CDSS recommendations when they conflict with their own clinical judgment or opinions [45,72,74]. These challenges impact the perceived validity and reliability of CDSS, influencing user trust and adoption [39,44,56,61,71,72].

However, emerging technologies such as XAI and context-aware machine learning systems offer promising solutions to address trust and validation challenges in CDSS. XAI enhances transparency by making AI systems’ inner workings more interpretable for health care professionals, enabling them to identify potential flaws and assess the trustworthiness of system outputs [83]. Additionally, context-aware machine learning systems that integrate patient-specific and environmental factors can generate more personalized and relevant recommendations, thereby reducing ambiguity and fostering trust in CDSS [84].

#### Data Quality or Assurance Issues

The effective design and implementation of machine learning and AI-enabled CDSS hinge fundamentally on the availability, accessibility, and quality of individual patient data, extracted ideally from their EHR. However, related challenges persist in this domain. A primary issue highlighted across the reviewed papers concerns access to quality data [47,53,67]. While vast clinical datasets may be available, navigating access protocols can be cumbersome due to data access privacy concerns [85,86].

Furthermore, existing data may lack curation or could be in formats that are unsuitable for CDSS integration [71]. Moreover, quality-related issues, such as errors and missing data, can arise during the process of transforming data into formats acceptable to CDSS, such as converting paper-based clinical records into digital data formats or converting images [41]. Other related challenges highlighted included interoperability issues, where different data standards used in the collection of medical data make their uptake by CDSS difficult [71]. Despite these obstacles, the adoption of EHRs and interoperability standards such as FHIR promises opportunities for improving data access and quality assurance in CDSS [87].

#### Design or Integration Complexities

The complexities inherent in the design of CDSS, coupled with integration challenges, present significant hurdles. Translating guidelines into decision rules that are implementable within CDSS presents nontrivial challenges [41,54,55], often requiring extensive data collection over extended periods.

Additionally, the diverse range of users adds another layer of complexity, particularly in designing input mechanisms that accommodate variability in user interactions [54,71]. For instance, designing a system capable of handling vague or incomplete user responses, which may occur with user input into CDSS, particularly in systems that do not support free-text entry CDSS, remains a major challenge. Moreover, modelling the clinical workflow for integration into the design of CDSS proves difficult due to the dynamic and constantly evolving nature of the clinical environment [51,71].

## Discussion

### Implications for Future Design of CDSS

This review provides a comprehensive analysis of CDSS design characteristics and challenges from a user-centered perspective. Key findings highlight the prevalent use of UCD and limitations in their integration with other clinical systems. Identified challenges encompass usability and UX, validity and reliability, data quality and assurance, and design and integration complexity.

This section discusses these principal findings and reflects on their implications for future CDSS design along 4 principal considerations: (1) Adopting a UCD approach in the iterative design process, (2) enhancing integration with health information systems, (3) leveraging XAI and human-in-the-loop techniques, and (4) implementing CDSS that support both clinical and patient-facing functions.

### Adopting a UCD Approach in the Iterative Design Process

The findings underscored a pervasive trend in CDSS design, where UCD principles are frequently adopted, albeit with varying levels of user engagement. Previous studies [11,14,17,88,89] corroborate the effectiveness of UCD in eliciting user needs, workflow requirements, and efficiency in CDSS design. Leveraging the strengths of UCD, particularly in comprehending user dynamics and design contexts, presents significant opportunities for mitigating identified challenges.

This review identified several usability and UX challenges, including workflow integration inconsistencies, covert surveillance [55], user fatigue [70], and support deficiencies [38,49,75], underscoring inefficiencies in end user engagement in the design process of most CDSS. These challenges can be effectively addressed by incorporating UCD in the design-testing-evaluation cycle. For example, engaging end users from the early stages of design, as emphasized in the UCD framework, facilitates the identification of workflow requirements. Additionally, the iterative usability testing and evaluation phases within UCD help detect early barriers to adoption, such as user fatigue and inadequate support structures.

Beyond usability, contextual challenges pertaining to technical complexities, such as translating guidelines into decision algorithms, technical implementation of workflow integration, and addressing the needs of different user groups [41,42,49,54], can be effectively navigated by adopting a UCD approach. Deep user engagement and stakeholder participation throughout the design process enable the identification and mitigation of contextual and human factors that may complicate the design, implementation, and use of CDSS. Furthermore, the iterative usability testing and evaluation stages within UCD provide substantial opportunities for user testing and refining technical features, such as decision algorithms, to ensure reliability, suitability, functionality, and appropriateness [14,15,34]. Effective user engagement and participation also foster a sense of empowerment and ownership [90,91], enhancing user understanding of the system’s operation and functionality, thereby addressing the trust and adoption-related concerns identified.

Effective user engagement and participation require that end users take a central role in the design and decision-making process. As such, it requires adopting conducive engagement strategies that foster collaboration, self-expression, and cocreation. Thus, the design of CDSS requires the adoption of methodologies and frameworks that prioritize deep user involvement and engagement. Design thinking approaches, particularly UCD, show unparalleled potential in this endeavor.

### Enhancing Integration With Digital Health Technologies

The findings highlight the limited integration of CDSS with other digital health technologies, which has significant implications for future CDSS design in a rapidly evolving digital health technology landscape. With the widespread adoption of emerging health care technologies and interoperability standards such as FHIR, integrating CDSS with digital health systems such as EHR and AI applications, including XAI, has become a critical focus. To ensure that these integrations meet user needs and expectations, it is essential to assess their impact on UX and adopt a user-centric approach from the outset to optimize implementation.

This review reveals data quality, access, and interoperability issues that pose significant challenges in accessing requisite and curated data, particularly for AI-enabled CDSS. Privacy concerns necessitate stringent legal protocols for patient data acquisition, while compatibility issues related to data formatting and extraction further complicate access for AI-driven CDSS [92-94]. Seamless integration with existing digital health technologies could enable CDSS to directly retrieve data from these technologies for learning, training, and modeling purposes.

Therefore, facilitating integration options between CDSS and existing digital health technologies presents opportunities for their effective implementation and functionality. For instance, integrating CDSS with personal health record (PHR) systems would allow direct retrieval of patient records, while AI-enabled CDSS could access data from these integrated systems for training, predictive modeling, and decision support [2,95]. Addressing the current limitations in CDSS integration with other health technologies is essential and warrants consideration as a fundamental requirement for future CDSS design.

Also, while acknowledging the complexities involved in accessing patient clinical data directly, particularly due to privacy and legal concerns, emerging interoperability standards such as FHIR offer avenues for mitigating some of them. For example, with FHIR, patient privacy through deidentification measures can be implemented, potentially fostering patients’ willingness to grant access to their deidentified clinical data for CDSS training and modeling purposes. Moreover, these interoperability standards formalize clinical data capture standards and formats, thereby alleviating challenges related to data formatting and curation [82,96].

Furthermore, using distributed architectures such as cloud computing and file-sharing applications can significantly improve CDSS design. These fault-tolerant and scalable systems, with decentralized components distributed across a network and coordinated through active communication, facilitate seamless interaction and coordination between independent components across various technologies. This approach enhances the overall efficacy and performance of the system, particularly in the context of integration [63,97,98].

### Implementing CDSS That Support Both Clinician-Facing and Patient-Facing Roles

A notable insight from this review is the predominant focus of CDSS on clinician-facing applications. Of the 40 systems analyzed, 37 were designed exclusively for clinical staff use. While this emphasis reinforces the critical role of CDSS in supporting clinical decision-making, it also limits their broader applicability in areas such as virtual care and integration with PHRs [99-101].

The current clinician-facing model requires patient’s presence for collecting and inputting vital signs and other clinical data. However, a dual-facing CDSS—designed for both clinician and patient use—could fundamentally transform this dynamic. By enabling patients to remotely input vital signs and other health metrics via a patient interface, such a system would allow clinicians to access, analyze, and provide recommendations through the same platform. This two-way functionality would enhance CDSS use in virtual care environments, facilitating seamless information exchange between patients and health care providers.

While implementing a dual-facing CDSS raises challenges related to access controls and network security, these issues can be effectively addressed through thoughtful software design and robust web-based connectivity solutions. Integrating CDSS into both clinician and patient workflows could significantly improve accessibility, streamline remote monitoring, and expand the role of CDSS in modern digital health care.

Considering the economic, accessibility, and efficiency benefits offered by technology-mediated health care options such as virtual care [102], CDSS present opportunities for adoption in such health care delivery schemes. For instance, the implementation of CDSS that facilitate the remote provision of patient vital signs (a requirement of most CDSS) could obviate the need for physical visits to medical facilities, thereby providing comfort and reducing health care delivery costs for both patients and health care providers [103].

However, this realization requires that CDSS support both clinician-facing and patient-facing capability to enable patients to provide such vital signs. A continuation of the prevailing trend, where they are predominantly clinician-facing, thwarts this opportunity. The current trend, where nearly all reviewed CDSS are tailored exclusively for clinicians, limits their broader applicability in virtual care settings. Notably, one of the few clinician-facing and patient-facing CDSS examined in this review [54] has demonstrated the advantages of virtual care and remote access, highlighting the feasibility and benefits of such an approach.

Furthermore, recent global health crises such as the COVID-19 pandemic, which rendered physical access to patients or clinicians impractical, underscore the importance of leveraging technology to mediate health care delivery that interfaces clinicians and patients. Implementing CDSS designed to accommodate both clinician and patient users would not only facilitate patient education but also encourage active participation in the health care process while promoting transparency. Such platforms can be strategically leveraged to educate patients on data ethics and address concerns that contribute to patient reluctance in allowing their data to be used for clinical purposes.

With the growing implementation and adoption of national health records across countries [2,104], PHR would become more accessible for leverage by patient-facing CDSS. This shift could significantly enhance health care delivery by fostering a more collaborative, patient-centered approach to clinical decision support.

### Harnessing XAI and Human-in-the-Loop Techniques in CDSS Design

A significant challenge highlighted across the reviewed papers relates to trust, validity, and reliability in adopting CDSS. The utilization of emerging technologies such as XAI [39,43,93,105] holds promise in addressing these challenges by ensuring human comprehension of AI decisions. XAI offers transparent explanations of the rationale behind the recommendations made by CDSS, thereby enhancing trust and reliability. This is buttressed by some of the studies reviewed in this study, which incorporated AI in their design to support explainability and trust, among other purposes [39,43,61,75].

Furthermore, incorporating human-in-the-loop techniques, which involve integrating end user participation in the decision-making process and capturing user assessment during their use for future training of the AI model, can enhance trust and transparency in CDSS [43,106]. For instance, providing users with options to select recommendations or request further explanations fosters human-AI collaboration and improves their acceptance and update of CDSS.

Thus, to foster trust, transparency, and adoption of CDSS, their design can leverage XAI, incorporate human-in-the-loop techniques, and use distributed systems architectures. These approaches integrate human input into the decision-making process of CDSS, enhancing trust and reliability while making CDSS more collaborative and suggestive.

### Limitations

We recognize that our review, which examined CDSS design through a UCD lens, may have overlooked certain technical implementation aspects, an area we plan to explore further in future research. While our focus was on design and UX, we did not delve deeply into technical considerations such as system architecture, algorithmic performance, or computational efficiency. As a result, our findings may not fully capture challenges related to system interoperability, algorithmic biases, or implementation scalability, which are critical from a technical standpoint.

However, by emphasizing design and UX factors, our review provides valuable insights into user adoption, usability challenges, and human-centered design principles, areas often underrepresented in purely technical evaluations. Additionally, despite conducting a comprehensive search across 5 major electronic libraries, some relevant literature may have been missed. Expanding the search scope to include a broader range of databases and keywords could have strengthened our findings. Furthermore, it is important to note that some of the CDSS discussed in the reviewed papers were not implemented or evaluated in clinical settings. As a result, potential real-world challenges and limitations may not have been fully explored.

### Conclusions

This review explored the design and challenges of CDSS. The findings highlight key design characteristics, including the frequent use of UCD approaches, a primary focus on clinician-facing designs, and integration gaps with other digital health technologies. The review identified 4 design challenges, including, usability and UX, validity and reliability, data quality and assurance, and design and integration complexities.

Drawing on these findings, several implications for future CDSS design were outlined. These include the need for more collaborative user engagement throughout the entire iterative design process, improved integration with other emerging digital health technologies, implementing CDSS that support both clinician-facing and patient-facing functions, and incorporating AI and human-in-the-loop architectures. These insights contribute to a deeper understanding of effective CDSS design, which is particularly relevant considering their increasing prominence in health care service delivery.

## Supplementary material

10.2196/63733 Multimedia Appendix 1 Search strategy.

10.2196/63733 Multimedia Appendix 2 Study characteristics.

10.2196/63733 Multimedia Appendix 3 List of included manuscripts.

10.2196/63733 Checklist 1 PRISMA (Preferred Reporting Items for Systematic Reviews and Meta-Analyses) checklist.

## Abbreviations

AI: artificial intelligence
CDSS: clinical decision support systems
EHR: electronic health record
GTLR: grounded theory literature review
MeSH: Medical Subject Headings
PHR: personal health record
PRISMA: Preferred Reporting Items for Systematic Reviews and Meta-Analyses
UCD: user-centered design
UX: user experience
XAI: explainable artificial intelligence
