# Correction: Designing Clinical Decision Support Systems (CDSS)—A User-Centered Lens of the Design Characteristics, Challenges, and Implications: Systematic Review

**DOI:** 10.2196/84380

**Published:** 2025-09-25

**Authors:** Andrew A Bayor, Jane Li, Ian A Yang, Marlien Varnfield

**Affiliations:** 1 Australian e-Health Research Centre, Commonwealth Scientific and Industrial Research Organisation, 296 Herston Rd, Herston, Brisbane, QLD, 4006, Australia, 61 7 3253 3620; 2 Thoracic Program, The Prince Charles Hospital, Brisbane, QLD, Australia; 3 Faculty of Medicine, The University of Queensland, Brisbane, QLD, Australia

In “Designing Clinical Decision Support Systems (CDSS)—A User-Centered Lens of the Design Characteristics, Challenges, and Implications: Systematic Review” [1], one sentence has been changed.

In the “Data Availability” section, the second sentence originally appeared as:

In addition, data on the search strategy, an overview of the findings, and author contributions are also attached as Multimedia Appendices 1, 3 and 4, respectively.

This sentence now reads:

In addition, data on the search strategy and an overview of the findings are also attached as Multimedia Appendices 1 and 2, respectively.

The correction will appear in the online version of the paper on the JMIR Publications website, together with the publication of this correction notice. Because this was made after submission to PubMed, PubMed Central, and other full-text repositories, the corrected article has also been resubmitted as needed to relevant repositories.
