# Supplementary material for: Designing Clinical Decision Support Systems (CDSS)—A User-Centered Lens of the Design Characteristics, Challenges, and Implications: Systematic Review
Source: J Med Internet Res. 2025 Jun 20;27:e63733. doi: 10.2196/63733 (PMC12463342; doi:10.2196/63733)
Supplement: Multimedia Appendix 1 [file jmir-v27-e63733-s001.pdf]

DATA SOURCES AND SEARCH STRATEGY

Inclusion Criteria:

- Article must be written in English.
- Article must be published between 2013 and 2023 (last 10 years).
- All article types are eligible, except reviews**,** systematic reviews or books.
- Article must be available in full text.
- Articles based on a design CDSS.
- Articles that describe either the design and user experience aspects of a CDSS such as its design approach, architecture, interfacing, or integration.

Exclusion Criteria:

- Articles that do not focus on a designed CDSS.
- Articles on CDSS interventions unrelated to health.
- Articles primarily focused on the technical implementation of CDSS.
- Conference abstracts and study protocols are excluded.
- Articles solely focusing on technical implementation or architecture (e.g., machine learning methods).

Search Keywords:

- Design
- User Experience
- Implementation
- Evaluation
- Usability
- Architecture

Databases Searched:

1. Journal of Decision Systems (Francis and Tailor)
2. PubMed
3. Scopus
4. Web of Science
5. IEEE Xplore

Search Query:

Database	Search Terms	No. of Articles
**PubMed**	1. ("Clinical Decision Support System*"[Title/Abstract]) AND ("Design"[Title/Abstract])<br>2. "Clinical Decision Support System*"[Title/Abstract]) AND ("User Experience"[Title/Abstract])<br>3. ("Clinical Decision Support System*"[Title/Abstract]) AND ("Implementation"[Title/Abstract])<br>4. ("Clinical Decision Support System*"[Title/Abstract]) AND ("Evaluation"[Title/Abstract])<br>5. ("Clinical Decision Support System*"[Title/Abstract]) AND ("Usability"[Title/Abstract])<br>6. ("Clinical Decision Support System*"[Title/Abstract]) AND ("Architecture"[Title/Abstract])<br>7. #1 OR #2 OR #3 OR #4 OR #5 OR #6	**279**
**Scopus**	TITLE ("Clinical Decision Support System*") AND TITLE-ABS-KEY("Design") OR TITLE-ABS-KEY("User Experience") OR TITLE-ABS-KEY("Implementation") OR TITLE-ABS-KEY("Evaluation") OR TITLE-ABS-KEY("Usability") OR Title-ABS-KEY("Architecture")	**625**
**Web of Science**	1. "Clinical Decision Support System*"(Title) AND "Design"(All Fields)<br>2. "Clinical Decision Support System*"(Title) AND "User Experience"(All Fields)<br>3. "Clinical Decision Support System*"(Title) AND "Implementation"(All Fields)<br>4. "Clinical Decision Support System*"(Title) AND "Evaluation"(All Fields)<br>5. "Clinical Decision Support System*"(Title) AND "Usability"(All Fields)<br>6. "Clinical Decision Support System*"(Title) AND "Architecture"(All fields)	**786**
**IEEE Xplore**	1. All: "Clinical Decision Support System*" AND "Design"<br>2. All: "Clinical Decision Support System*" AND "User Experience"<br>3. All: "Clinical Decision Support System*" AND "Implementation"<br>4. All: "Clinical Decision Support System*" AND "Evaluation"<br>5. All: "Clinical Decision Support System*" AND "Usability"<br>6. All: "Clinical Decision Support System*" AND "Architecture"	**215**
	Total	**1905**
