# Supplementary material for: Designing Clinical Decision Support Systems (CDSS)—A User-Centered Lens of the Design Characteristics, Challenges, and Implications: Systematic Review
Source: J Med Internet Res. 2025 Jun 20;27:e63733. doi: 10.2196/63733 (PMC12463342; doi:10.2196/63733)
Supplement: Multimedia Appendix 3 [file jmir-v27-e63733-s003.pdf]

List of 40 Included papers in the Review

1. Abtahi H, Amini S, Gholamzadeh M, Gharabaghi MA. Development and evaluation of a mobile-based asthma clinical decision support system to enhance evidence-based patient management in primary care. Informatics in Medicine Unlocked 2023;37:101168. doi: 10.1016/j.imu.2023.101168

2. Alhodaib HI, Antza C, Chandan JS, Hanif W, Sankaranarayanan S, Paul S, Sutcliffe P, Nirantharakumar K. Mobile Clinical Decision Support System for the Management of Diabetic Patients With Kidney Complications in UK Primary Care Settings: Mixed Methods Feasibility Study. JMIR Diabetes 2020 Nov 18;5(4):e19650. doi: 10.2196/19650

3. Barigou et al. The Design of a Cloud-based Clinical Decision Support System Prototype: Management of Drugs Intoxications in Childhood. International Journal of Healthcare Information Systems and Informatics.

4. Beerlage-de Jong N, Wentzel J, Hendrix R, van Gemert-Pijnen L. The value of participatory development to support antimicrobial stewardship with a clinical decision support system. American Journal of Infection Control 2017 Apr 1;45(4):365–371. doi: 10.1016/j.ajic.2016.12.001

5. Garbern SC, Nelson EJ, Nasrin S, Keita AM, Brintz BJ, Gainey M, Badji H, Nasrin D, Howard J, Taniuchi M, Platts-Mills JA, Kotloff KL, Haque R, Levine AC, Sow SO, Alam NH, Leung DT. External validation of a mobile clinical decision support system for diarrhea etiology prediction in children: A multicenter study in Bangladesh and Mali. Schiffer JT, Serwadda DM, Kanjilal S, editors. eLife eLife Sciences Publications, Ltd; 2022 Feb 9;11:e72294. doi: 10.7554/eLife.72294

6. Gaudaen JC, Papadopoulos A, Dockery T, Manemeit C. Usability Enhancements to a Prototype Clinical Decision Support System for Combat Medics. Military Medicine 2023 Nov 8;188(Supplement_6):614–620. doi: 10.1093/milmed/usad279

7. Hosseini A, Asadi F, Arani LA. Development of a Knowledge-based Clinical Decision Support System for Multiple Sclerosis Diagnosis. J Med Life 2020;13(4):612–623. PMID:33456613

8. Kemppinen J, Korpela J, Elfvengren K, Salmisaari T, Polkko J. Decision Support in Evaluating the Impacts of Mental Disorders on Work Ability. 2014 47th Hawaii International Conference on System Sciences 2014. p. 2958–2966. doi: 10.1109/HICSS.2014.368

9. Kieffer S, Gouze A, Vanderdonckt J. A Multimodal, Usable, and Flexible Clinical Decision-Support System for Breast Cancer Diagnosis and Reporting. SN COMPUT SCI 2022 Nov 11;4(1):49. doi: 10.1007/s42979-022-01451-z

10. Knight SR, Cao KN, South M, Hayward N, Hunter JP, Fox J. Development of a Clinical Decision Support System for Living Kidney Donor Assessment Based on National Guidelines. Transplantation 2018 Oct;102(10):e447. doi: 10.1097/TP.0000000000002374

11. Kropf M, Modre-Osprian R, Hayn D, Fruhwald F, Schreier G. Telemonitoring in heart failure patients with clinical decision support to optimize medication doses based on guidelines. 2014 36th Annual International Conference of the IEEE Engineering in Medicine and Biology Society Chicago, IL: IEEE; 2014. p. 3168–3171. doi: 10.1109/EMBC.2014.6944295

12. López MM, López MM, de la Torre Díez I, Jimeno JCP, López-Coronado M, Rodrigues JJPC. Evaluating the QoE of a mobile DSS for diagnosis of red eye diseases by medical students. 2016 IEEE 18th International Conference on e-Health Networking, Applications and Services (Healthcom) 2016. p. 1–6. doi: 10.1109/HealthCom.2016.7749498

13. Luna D, Otero C. Redesign of a clinical decision support system for a drug - drug interaction alert (Extended: User-centered design improves the usability of drug-drug interaction alerts: Experimental comparison of interfaces). DOI: 10.1016/j.jbi.2017.01.009

14. Michot E, Woo J, Mouline L, Sinnappan C, Boukobza A, Campeotto F, Dupic L, Burgun A, Vivien B, Tsopra R. Towards a Clinical Decision Support System for Helping Medical Students in Emergency Call Centers. In: Séroussi B, Weber P, Dhombres F, Grouin C, Liebe J-D, Pelayo S, Pinna A, Rance B, Sacchi L, Ugon A, Benis A, Gallos P, editors. Studies in Health Technology and Informatics IOS Press; 2022. doi: 10.3233/SHTI220494ISBN:978-1-64368-284-6

15. Nair KM, Malaeekeh R, Schabort I, Taenzer P, Radhakrishnan A, Guenter D. A Clinical Decision Support System for Chronic Pain Management in Primary Care: Usability testing and its relevance. jhi 2015 Aug 13;22(3):329–332. doi: 10.14236/jhi.v22i3.149

16. Nasir IS, Mousa AH, Ali Alkhafaji SM, Abdul Hussein WS, Jasim ZR, Ali SQ. Virtual data integration for a clinical decision support systems. IJECE 2023 Oct 1;13(5):5243. doi: 10.11591/ijece.v13i5.pp5243-5252

17. Panigutti C, Beretta A, Fadda D, Giannotti F, Pedreschi D, Perotti A, Rinzivillo S. Co-design of Human-centered, Explainable AI for Clinical Decision Support. ACM Trans Interact Intell Syst 2023 Dec 31;13(4):1–35. doi: 10.1145/3587271

18. PAYDAR P, EBRAHIMPOUR S, ZEHTAB HASHEMI H, MOHAMADI M, NAMAZI S. Design, Development and Evaluation of an Application based on Clinical Decision Support Systems (CDSS) for Over-The-Counter (OTC) Therapy: An Educational Interventions in Community Pharmacists. J Adv Med Educ Prof 2023 Apr;11(2):95–104. PMID:37113680

19. Ray JM, Ahmed OM, Solad Y, Maleska M, Martel S, Jeffery MM, Platts-Mills TF, Hess EP, D'Onofrio G, Melnick ER. Computerized Clinical Decision Support System for Emergency Department–Initiated Buprenorphine for Opioid Use Disorder: User-Centered Design. JMIR Hum Factors 2019 Feb 27;6(1):e13121. doi: 10.2196/13121

20. Seitinger A, Fehre K, Adlassnig K-P, Rappelsberger A, Wurm E, Aberer E, Binder M. An Arden-Syntax-based clinical decision support framework for medical guidelines--Lyme borreliosis as an example. Studies in health technology and informatics 2014 May 14;198:125–32.

21. Shahmoradi L, Safdari R, Mirhosseini MM, Rezayi S, Javaherzadeh M. Development and evaluation of a clinical decision support system for early diagnosis of acute appendicitis. Sci Rep Nature Publishing Group; 2023 Nov 11;13(1):19703. doi: 10.1038/s41598-023-46721-9

22. Shalom E, Goldstein A, Ariel E, Sheinberger M, Jones V, Van Schooten B, Shahar Y. Distributed application of guideline-based decision support through mobile devices: Implementation and evaluation. Artif Intell Med 2022 Jul;129:102324. PMID:35659389

23. Silva EAT, Gomez IFL, Arango JFF, Smith JW, Ocampo SU, Hidalgo JE. EVALUATION OF SATISFACTION AND USABILITY OF A CLINICAL DECISION SUPPORT SYSTEM (CDSS) TARGETED FOR EARLY OBSTETRIC RISK ASSESSMENT AND PATIENT FOLLOW-UP. 2018;

24. Tanguay-Sela M, Benrimoh D, Popescu C, Perez T, Rollins C, Snook E, Lundrigan E, Armstrong C, Perlman K, Fratila R, Mehltretter J, Israel S, Champagne M, Williams J, Simard J, Parikh SV, Karp JF, Heller K, Linnaranta O, Cardona LG, Turecki G, Margolese HC. Evaluating the perceived utility of an artificial intelligence-powered clinical decision support system for depression treatment using a simulation center. Psychiatry Research 2022 Feb 1;308:114336. doi: 10.1016/j.psychres.2021.114336

25. Tarnowska KA, Dispoto BC, Conragan J. Explainable AI-based clinical decision support system for hearing disorders. AMIA Jt Summits Transl Sci Proc 2021 May 17;2021:595–604. PMID:34457175

26. Ulapane N, Forkan ARM, Jayaraman PP, Schofield P, Burbury K, Wickramasinghe N. Using Task Technology Fit Theory to Guide the Codesign of Mobile Clinical Decision Support Systems.

27. Vogel S, Reiswich A, Ritter Z, Schmucker M, Fuchs A, Pischek-Koch K, Wache S, Esslinger K, Dietrich M, Kesztyüs T, Krefting D, Haag M, Blaschke S. Development of a Clinical Decision Support System for Smart Algorithms in Emergency Medicine. In: Mantas J, Hasman A, Househ MS, Gallos P, Zoulias E, Liaskos J, editors. Studies in Health Technology and Informatics IOS Press; 2022. doi: 10.3233/SHTI210900ISBN:978-1-64368-250-1

28. Wright A, Sittig DF, Ash JS, Erickson JL, Hickman TT, Paterno M, Gebhardt E, McMullen C, Tsurikova R, Dixon BE, Fraser G, Simonaitis L, Sonnenberg FA, Middleton B. Lessons learned from implementing service-oriented clinical decision support at four sites: A qualitative study. Int J Med Inform 2015 Nov;84(11):901–911. PMID:26343972

29. Yin Z, Dong Z, Lu X, Yu S, Chen X, Duan H. A clinical decision support system for the diagnosis of probable migraine and probable tension-type headache based on case-based reasoning. J Headache Pain 2015 Apr 1;16(1):29. doi: 10.1186/s10194-015-0512-x

30. Zhao Y, Hu J, Gu Y, Wan Y, Liu F, Ye C, Zhang X. Development and Implementation of a Pediatric Nursing-Clinical Decision Support System for Hyperthermia. Comput Inform Nurs 2021 Aug 4;40(2):131–137. PMID:34347639

31. Zhang X, Svec M, Tracy R, Ozanich G. Clinical decision support systems with team-based care on type 2 diabetes improvement for Medicaid patients: A quality improvement project. International Journal of Medical Informatics 2022 Feb 1;158:104626. doi: 10.1016/j.ijmedinf.2021.104626

32. Anakal SS, Sandhya P. Clinical Decision Support System for Diagnosis and Treatment of COPD Using Ensemble Methods. Stud Comput Intell Springer Science and Business Media Deutschland GmbH; 2023. p. 1–23. doi: 10.1007/978-981-99-1482-1_1ISBN:1860949X (ISSN)

33. Breitbart E, Choudhury K, Andersen A, Bunde H, Breitbart M, Sideri A, Fengler S, Zibert J. Improved patient satisfaction and diagnostic accuracy in skin diseases with a Visual Clinical Decision Support System-A feasibility study with general practitioners. PLOS ONE 2020 Jul 29;15(7). doi: 10.1371/journal.pone.0235410

34. Conway N, Adamson KA, Cunningham SG, Emslie Smith A, Nyberg P, Smith BH, Wales A, Wake DJ. Decision Support for Diabetes in Scotland: Implementation and Evaluation of a Clinical Decision Support System. J Diabetes Sci Technol SAGE Publications Inc.; 2018;12(2):381–388. doi: 10.1177/1932296817729489

35. Farmer N. An update and further testing of a knowledge-based diagnostic clinical decision support system for musculoskeletal disorders of the shoulder for use in a primary care setting. JOURNAL OF EVALUATION IN CLINICAL PRACTICE 2014 Oct;20(5):589–595. doi: 10.1111/jep.12153

36. Firzara A, Teo C, Teh S, Su J, Zaini H, Suhaimi A, Ng W, Danaee M, Stevenson K, Mallen C, Ng C. Evaluation of an electronic clinical decision support system (DeSSBack) to improve low back pain management: a pilot cluster randomized controlled trial. FAMILY PRACTICE 2023 May 27; doi: 10.1093/fampra/cmad044

37. Khong PCB, Lee LN, Dawang AI. Modeling the construct of an expert evidence-adaptive knowledge base for a pressure injury clinical decision support system. Informatics MDPI AG; 2017;4(3). doi: 10.3390/informatics4030020

38. Segal G, Segev A, Brom A, Lifshitz Y, Wasserstrum Y, Zimlichman E. Reducing drug prescription errors and adverse drug events by application of a probabilistic, machine-learning based clinical decision support system in an inpatient setting. J Am Med Informatics Assoc Oxford University Press; 2019;26(12):1560–1565. doi: 10.1093/jamia/ocz135

39. Sim L, Ban K, Tan T, Sethi S, Loh T. Development of a clinical decision support system for diabetes care: A pilot study. PLOS ONE 2017 Feb 24;12(2). doi: 10.1371/journal.pone.0173021

40. Steffny L, Dahlem N, Reichl L, Gisa K, Greff T, Werth D. Design of a Human-in-the-Loop Centered AI-Based Clinical Decision Support System for Professional Care Planning. In: Lukowicz P., Mayer S., Koch J., Shawe-Taylor J., Tiddi I., editors. Front Artif Intell Appl IOS Press BV; 2023. p. 263–273. doi: 10.3233/FAIA230090
